# Use of Intraoperative Ultrasonography of the Small Bowel to Reduce Histologically Positive Margins in Crohn’s Disease Surgery: A Pilot Study

**DOI:** 10.3390/jcm14093135

**Published:** 2025-04-30

**Authors:** Franco Sacchetti, Fabrizio Pizzolante, Mauro Giambusso, Carmen Nesci, Diana Giannarelli, Federica Galiandro, Daniela Pugliese, Franco Scaldaferri, Maria C. Giustiniani, Domenico Balzano, Paola Caprino, Angelo E. Potenza, Laura M. Minordi, Luigi Sofo

**Affiliations:** 1 UOC di Chirurgia Addominale, Dipartimento di Scienze Gastroenterologiche, Endocrino-Metaboliche e Nefro-Urologiche, Fondazione Policlinico Universitario A. Gemelli IRCCS, 00136 Rome, Italy; franco.sacchetti@policlinicogemelli.it (F.S.); federica.galiandro@policlinicogemelli.it (F.G.); paola.caprino@policlinicogemelli.it (P.C.); angeloeugenio.potenza@policlinicogemelli.it (A.E.P.); luigi.sofo@policlinicogemelli.it (L.S.); 2 UOC CEMAD, Centro Malattie dell’Apparato Digerente, Fondazione Policlinico Universitario A. Gemelli IRCCS, 00136 Rome, Italy; fabrizio.pizzolante@policlinicogemelli.it (F.P.); daniela.pugliese@policlinicogemelli.it (D.P.); franco.scaldaferri@policlinicogemelli.it (F.S.); 3 Scuola di Specializzazione in Chirurgia Generale, Università Cattolica del Sacro Cuore, 00168 Rome, Italy; maurogiambusso@gmail.com (M.G.); carmennesci96@gmail.com (C.N.); domenico.94.balzano@gmail.com (D.B.); 4 Divisione di Chirurgia Generale, Ospedale Vittorio Emanuele, 93012 Gela, Italy; 5 Facility di Epidemiologia e Biostatistica, Fondazione Policlinico Universitario A. Gemelli IRCCS, 00136 Rome, Italy; diana.giannarelli@policlinicogemelli.it; 6 Dipartimento di Medicina e Chirurgia Traslazionale, Università Cattolica del Sacro Cuore, 00168 Rome, Italy; 7 UOS di Gastroenterologia, Ospedale Isola Tiberina Gemelli Isola, 00186 Rome, Italy; 8 Dipartimento di Patologia, Fondazione Policlinico Universitario A. Gemelli IRCCS, 00136 Rome, Italy; mariacristina.giustiniani@policlinicogemelli.it; 9 Dipartimento di Diagnostica per Immagini, Radioterapia Oncologica ed Ematologia, Fondazione Policlinico Universitario A. Gemelli IRCCS, L.go A. Gemelli 8, 00136 Rome, Italy

**Keywords:** Crohn’s disease, intraoperative ultrasound, postoperative recurrence, IBD

## Abstract

**Background/Objectives**: The histological involvement of surgical resection margins in Crohn’s disease (CD) is an important risk factor for postoperative recurrence. The aim of this study was to evaluate the usefulness of intraoperative ultrasonography (IOUS) of the small bowel to best identify the surgical site of resection and reduce the rate of the histological involvement of margins. **Methods**: Consecutive patients who underwent ileocolic surgery for CD were prospectively enrolled (IOUS group) and underwent IOUS to fix the resection site. A control historical group of patients undergoing the same surgical procedures was considered and a 1:1 propensity score matching for location of disease and repeated surgery was performed. The primary endpoint was the histological involvement of resection margins. The secondary endpoint was to assess the feasibility of the method. **Results**: Twenty-seven patients were enrolled in the IOUS group and twenty-seven were enrolled in the non-IOUS group. The two groups were homogeneous in terms of gender, age, smoking, BMI, behavior of disease, and surgical technique. The IOUS group presented a lower rate of histological positive margins (18.5% vs. 48.1%; *p* = 0.021). No significant differences were found in terms of mean duration of surgery (IOUS: 254.2 min vs. non-IOUS: 225 min [SD = 49.3–77.8]; *p* = 0.11) or in terms of mean length of surgical specimen (IOUS: 24.1 cm vs. non-IOUS: 34.1 cm [SD = 13.5–23.1]; *p* = 0.058). **Conclusions**: IOUS of the small bowel appears to be a useful tool to obtain a lower rate of histologically positive margins with a comparable duration of surgery and no significant difference in the intestinal specimen length.

## 1. Introduction

Crohn’s disease (CD) is becoming more common in Western countries, and newly industrialized and developing nations are also experiencing a significant increase in its incidence [1]. Despite advancements in medical therapy, more than half of patients with CD will require surgery during their lifetime. However, surgery is not curative, and approximately 40% of patients require additional surgical intervention within 10 years due to postoperative recurrence (POR) [2].

POR may manifest endoscopically, clinically, radiologically, or surgically. Its occurrence is associated with several risk factors related to the patient (e.g., sex, age, smoking habits) [3], the disease itself (e.g., fistulizing phenotype, disease duration, or concomitant perianal involvement), and surgical factors. Among the latter, the presence of microscopic signs of CD—such as granulomas, ulcers, and serositis—at the resection margins appears to play a crucial role [4]. Based on the available evidence, the European Crohn’s and Colitis Organisation (ECCO) has included the presence of granulomas and inflammation of the myenteric plexus at the resection margins as risk factors for POR in its most recent guidelines [5,6].

Currently, the site of intestinal resection is decided based on the absence of macroscopic signs of inflammation by the surgeon’s evaluation.

Recently, some authors proposed the use of intraoperative ultrasound (IOUS) of the small bowel to better localize pathological segments during surgery.

This method has been shown to have high sensitivity and accuracy in identifying CD locations when compared with percutaneous ultrasonography and magnetic resonance imaging [7]. At present, there is no recommendation on the routine use of IOUS in clinical practice. The aim of our study is to evaluate the usefulness of IOUS in accurately identifying the surgical site of proximal resection and reducing the risk of the histological involvement of resection margins.

## 2. Materials and Methods

### 2.1. Study Design

A single-center prospective interventional study was conducted, enrolling consecutive patients affected by CD and undergoing ileocecal or ileocolic resections for first or repeated surgery at the Abdominal Surgery Unit of the Fondazione Policlinico Universitario A. Gemelli IRCCS from April 2022 to April 2023. The study protocol was approved by the Ethics Committee of Fondazione Policlinico Universitario A. Gemelli IRCCS, Università Cattolica del Sacro Cuore, Rome, Italy (ID: 5836; ClinicalTrials.gov number: NCT06388057). Patients included in the study provided written informed consent for their clinical data to be used for research purposes.

### 2.2. Intraoperative Ultrasound

**Figure 1:**
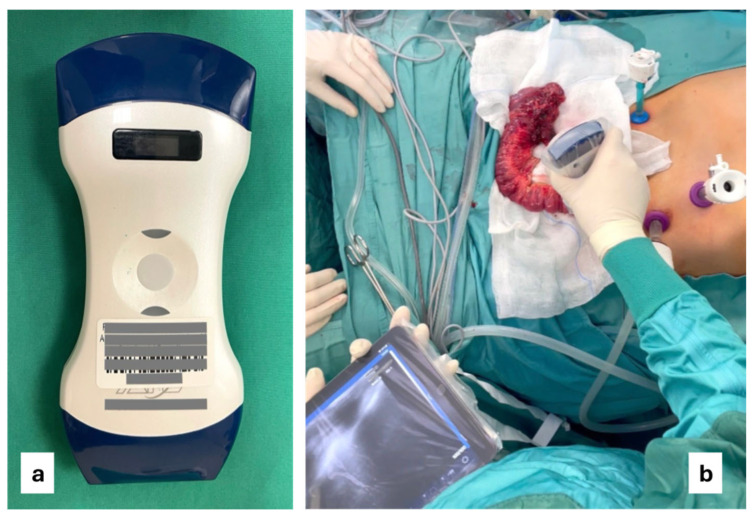
Wireless probe used in IOUS (**a**); IOUS during ileocecal resection for Crohn’s disease (**b**).

All patients underwent intraoperative ultrasound with a wireless linear probe [Figure 1a] performed by a dedicated gastroenterologist expert in inflammatory bowel disease ultrasonography [Figure 1b].

**Figure 2:**
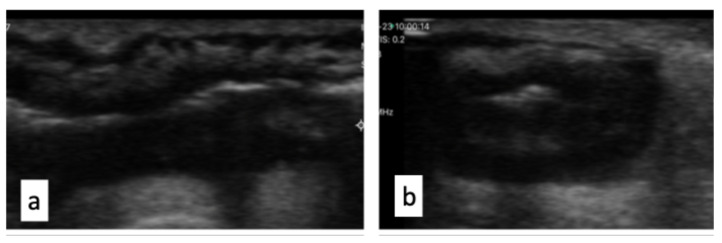
Longitudinal section (**a**) and transverse section (**b**) of ileal loop: wall appears thickened, hypoechogenic, and lacking physiological stratification.

**Figure 3:**
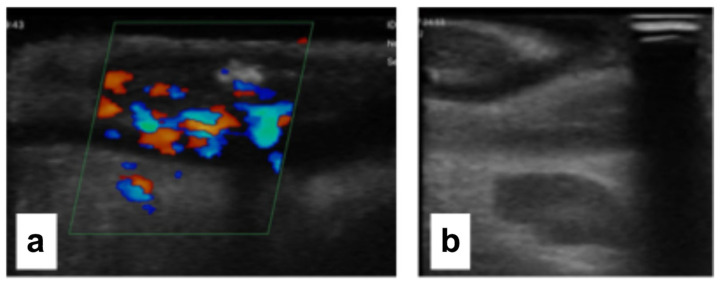
Visible transmural vascular signals are observed on color Doppler imaging (**a**), and a reactive lymph node (**b**) is discernible in the context of the mesentery in active Crohn’s disease.

For these patients, the site of the small bowel resection was decided considering both the absence of macroscopic (absence of wall thickening, creeping fat, or wall hyperemia) and ultrasonographic signs of CD (the IOUS group). Ultrasonographic activity was defined by the presence of a bowel wall thickening (BWT) > 3 mm and bowel wall pattern (BWP) as previously reported in the literature [8] [Figure 2 and Figure 3].

None of the enrolled patients had colonic disease, so the choice of the site of colonic resection was not an object of this study.

### 2.3. Data Collection

The following baseline characteristics were recorded at enrolment: sex, age, body mass index (BMI), smoking status, stenosing or fistulizing disease phenotype, location of disease (ileocecal or anastomotic), concomitant perianal disease, history of previous surgery, and surgical approach of the index surgery (open or laparoscopic).

A control group from the historical cohort of patients who underwent the same surgical procedures between January 2017 and April 2022 was considered; for this group, the site of small bowel resection was decided solely on the absence of macroscopic signs of disease as previously mentioned.

In order to reduce possible bias related to the non-homogeneity of the groups, propensity score matching was performed, and the analysis identified a subgroup of patients in this cohort (non-IOUS group) matched to the IOUS group on the basis of disease location and surgery for recurrence and homogeneous in terms of gender, age, smoking, BMI, disease pattern, and surgical technique (laparoscopic vs. open).

Each surgical procedure performed in both patient groups was carried out by only two surgeons, and no significant modifications were made to the surgical technique throughout the study period.

### 2.4. Endpoint

**Figure 4:**
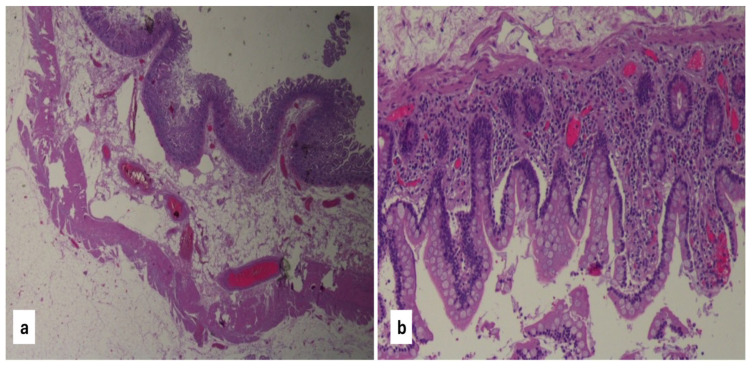

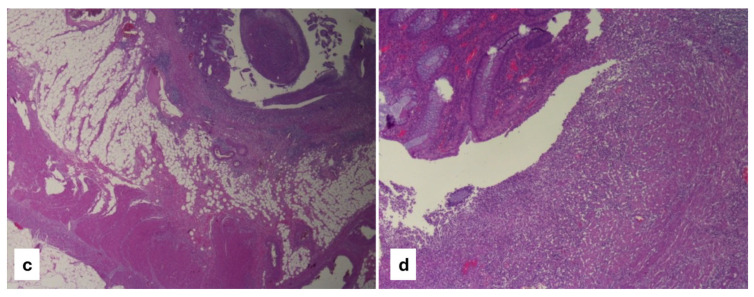
Surgical resection margin free from histological signs of Crohn’s disease (**a**,**b**). Resection margin involved by Crohn’s disease with the presence of deep ulcers and granulomas (**c**,**d**).

The primary endpoint of our study was to verify whether the use of IOUS could reduce the rate of histological positivity of the surgical resection margins. Specimen preparation and microscopic analysis were performed by two pathologists experienced in IBD. The resection margins were considered histologically positive in the presence of acute inflammatory lesions, including erosion, ulceration, and infiltration of the chorion by neutrophil, cryptic abscesses, or cryptitis lesions [Figure 4a–d].

The secondary endpoint was to demonstrate the feasibility of the IOUS method, namely, exploring the impact of IOUS on the length of the surgical piece and the duration of surgery.

### 2.5. Statistical Analysis

Continuous data were summarized with means and standard deviations (SDs), while categorical data were presented with absolute frequencies and percentages. Differences between the study groups were assessed according to Student’s t-tests and chi-square tests, as appropriate.

Univariate linear logistic regression was used to test the correlation between the risk factors and the analyzed outcomes. To correct for confounding factor bias and minimize differences between groups, a propensity score matching analysis was introduced, matching patients 1:1 for disease location and repeated surgery and calculated according to K-Nearest Neighbors models without replacement and with a 0.1 SD distribution gauge. Statistical significance was set at *p* < 0.05. Statistical analyses were conducted using IBM SPSS Statistics (v. 27.0) and R software (v. 4.1.2, MatchIt library).

## 3. Results

### 3.1. Patient Characteristics

Twenty-seven patients (16 males and 11 females, mean age = 38.3 years, SD = 16.5) were enrolled (IOUS group). The mean BMI was 21.2 kg/m^2^ (SD = 2). Five of the twenty-seven subjects (18.5%) were smokers. The disease pattern was stenosing in 12 cases (44.4%) and fistulizing in 14 cases (51.9%). Only one patient (3.7%) had a history of concomitant perianal disease. Regarding CD location, 8 patients (29.6%) had an ileal location and 19 (70.4%) had an ileocolic location. The surgical approach was laparoscopic in 18 of the cases (66.7%). Seven patients (25.9%) underwent surgery for disease recurrence. The historical cohort included 205 patients, of which 96 were female (46.8%), with a mean age of 42.5 years (SD = 15.7) [Table 1].

### 3.2. Results of the Statistical Analysis

After propensity score matching, 27 patients (non-IOUS group) were identified, of which 12 were female (44.4%), with a mean age of 40.4 years (SD = 15.2). The mean BMI was 20.6 (SD = 5.1). Seven patients were smokers (25.9%). The stenosing phenotype of disease was present in 16 cases (59.3%), penetrating in 11 patients (40.7%). The site of disease was ileal in 29.6% of cases (8 patients) and ileocolic in 70.4% of cases (19 patients). Coexisting perianal disease was detected in seven patients (25.9%). Surgery was performed laparoscopically in 48.1% of cases (13 patients). Surgery for recurrence was performed in seven patients (25.9%) [Table 2]. The IOUS group presented a statistically significant lower rate of positive histological margins compared to the non-IOUS group (5 [18.5%] vs. 13 patients [48.1%]; *p* = 0.021). Furthermore, no significant differences were found between the IOUS and non-IOUS groups in terms of mean duration of surgery (254.2 min [SD = 49.3] vs. 225 min [SD = 77.8]; *p* = 0.11] or in terms of mean length of surgical resection (24.1 cm [SD = 13.5] vs. 34.1 cm [SD = 23.1]; *p* = 0.058) [Table 3].

## 4. Discussion

POR of CD is a relevant problem that affects a significant percentage of patients suffering from this pathology. Several risk factors have been implicated in the development of postoperative recurrence (POR) in Crohn’s disease, including genetic susceptibility, disease-related features (such as immune-mediated mechanisms, the presence of perianal disease, and a penetrating or fistulizing phenotype), patient-related behaviors (notably smoking), and surgical variables [9]. Among the surgical factors, one of the most consistently reported is the presence of microscopic Crohn’s disease at the resection margins, identified in approximately 32% to 40% of cases. This histological finding has been linked to a higher risk of POR [4,10,11].

Notably, a retrospective study conducted by the Leuven and Amsterdam centers in 2017, which included 538 patients who underwent a first ileocecal resection for Crohn’s disease between 1998 and 2013, demonstrated that microscopic involvement of the resection margins was an independent risk factor for surgical recurrence (HR = 2.99; 95% CI = 1.36–6.54; *p* = 0.006) [12]. Similarly, in 2020, Poredska et al. reported in a case series of 107 patients operated on for Crohn’s disease between 2012 and 2018 that histological evidence of disease at the resection margins was significantly associated with higher rates of endoscopic recurrence at 6 months (56.5% vs. 4.8%; *p* < 0.001) [13]. The number of scientific studies grew to such an extent that Tandon et al. [14] in 2021 conducted a systematic review and meta-analysis encompassing 21 studies and a total of 2481 patients with Crohn’s disease. Their analysis demonstrated that positive resection margins were associated with an increased risk of both clinical (RR = 1.26; 95% CI = 1.06–1.49; I^2^ = 41%) and surgical (RR = 1.87; 95% CI = 1.14–3.08; I^2^ = 71%) POR.

More recently, in 2023, Yzet et al. [6] from Paris published another systematic review and meta-analysis including thirty studies: seven focused on myenteric plexitis, six on submucosal plexitis, and twenty-three on positive margins.

The conclusions of the meta-analysis reported that inflammatory margins were associated with a higher rate of clinical (OR = 2.38; 95% CI = 1.54–3.68) and surgical recurrences (OR = 1.52; 95% CI = 1.07–2.16). Additionally, the presence of myenteric plexitis was associated with a higher rate of clinical and endoscopic recurrence. Whatever the current motivation, reducing the number of positive margins is important to identify those patients at high risk of recurrence who may benefit from early prophylactic pharmacological treatment, as indicated by current guidelines [15].

Intraoperative strategies aimed at reducing the rate of histologically positive resection margins cannot include extemporaneous biopsies, as they cannot be considered reliable or reproducible due to their high costs and time requirements [16]. The use of ultrasound technology is now widespread and recognized as a valid diagnostic technique for identifying the presence and extent of CD [17].

In 2019, Viganò et al. [7] demonstrated that intraoperative ultrasonography of the small bowel had higher levels of sensitivity and specificity in staging and identifying CD compared to preoperative transabdominal ultrasonography and magnetic resonance imaging (MRI), while also allowing the identification of additional skip lesions. In the same year, Celentano et al. [18] developed an intraoperative protocol (the Portsmouth protocol) to standardize the IOUS technique for Crohn’s disease, demonstrating that the method, in addition to being sensitive and specific in identifying CD, can be a valuable support for surgeons by providing important information and, sometimes, modifying the surgical strategy [19].

In our study, the use of IOUS allowed a significantly lower rate of histologically positive margins to be obtained. Furthermore, we observed a numerically higher rate in the length of the surgical specimen (almost 10 cm on average) in the non-IOUS group compared to the group that underwent intraoperative ultrasound. Moreover, even though statistical significance was not reached, the non-IOUS group also showed a shorter average duration of surgical intervention (almost 30 min), which is comparable to previous experience [19].

As in the study by Celentano et al. [18], the IOUSs reported in our paper were performed by a gastroenterologist with expertise in IBD. While this aspect may limit the reproducibility of the procedure, it highlights the importance of performing this type of surgery in referral centers equipped to provide multidisciplinary management of patients. In this regard, it is essential to clarify that the use of intraoperative ultrasound (IOUS) proposed in this pilot study is not intended to identify or thoroughly characterize the segments affected by Crohn’s disease. Rather, its sole purpose is to detect the suspected presence of disease at the site selected for surgical resection. Within this framework, we believe that IOUS cannot currently replace a thorough preoperative imaging assessment (CT or MRI), which remains crucial for accurately evaluating the extent and localization of the disease and for guiding the overall surgical strategy [20]. That said, we cannot conceal our enthusiasm for the use of ultrasound in the management of Crohn’s disease. In selected scenarios, the use of laparoscopic or robotic ultrasound probes allows the examination to be performed entirely intracorporeally through a minimally invasive approach, thereby reducing surgical trauma.

Our study represents a pilot experience, inherently characterized by several limitations, primarily related to the small sample size and the use of a historical control group.

Although the use of propensity score matching improved the homogeneity between the two groups, it is important to acknowledge that comparisons with a historical cohort are inherently subject to historical control bias, which cannot be entirely eliminated.

These limitations prevent us from drawing definitive conclusions regarding the value of IOUS in reducing histologically positive margins and, consequently, in the indirect reduction of postoperative recurrence (POR). To validate these preliminary findings and evaluate their impact on long-term POR rates and cost-effectiveness, larger prospective, multicenter randomized controlled trials are essential, despite the encouraging initial results. Nonetheless, these promising outcomes have opened new perspectives and prompted the initiation of a prospective trial (ClinicalTrials.gov identifier: NCT06388057), which is currently in the recruitment phase at our center. This study aims to compare the traditional macroscopic criterion with an ultrasound-based approach for identifying the optimal surgical resection site, with the goal of generating robust scientific evidence to further evaluate the potential of this technique. Within this framework, the study will also directly investigate the role of IOUS in the postoperative recurrence of Crohn’s disease with long-term follow-up.

## 5. Conclusions

IOUS of the small bowel appears to be a useful tool for guiding the IBD surgeon in defining the best intestinal resection site to reduce the rate of histologically positive margins. This technique has proven to be well reproducible, cheap, and feasible without significant increases in the duration of the surgery or in the length of the surgical specimen. Prospective randomized studies conducted with high methodological standards are necessary to corroborate these preliminary results.

## Figures and Tables

**Table 1:** Characteristics of patients in the historical cohort and IOUS groups.

KERRYPNX	Historical Cohort (*n* = 205)	IOUS (*n* = 27)	*p* Value
**Gender**			0.55
F	109 (53.2)	16 (59.3)	
M	96 (46.8)	11 (40.7)	
**Age (years)**			
Mean (SD)	42.5 (15.7)	38.3 (16.5)	0.19
**BMI**			
Mean (SD)	21.90 (4.14)	21.24 (2.03)	0.42
**Smoking**			
No	145 (70.7)	22 (81.5)	
Yes	60 (29.3)	5 (18.5)	0.24
**Stricturing disease**			
No	76 (37.1)	15 (55.6)	
Yes	129 (62.9)	12 (44.4)	0.064
**Penetrating disease**			
No	102 (49.8)	13 (48.1)	
Yes	103 (50.2)	14 (51.9)	0.87
**Localization**			
Ileal	62 (30.2)	8 (29.6)	
Ileocolonic	143 (69.8)	19 (70.4)	0.95
**Perianal disease**			
No	166 (81.0)	26 (96.3)	
Yes	39 (19.0)	1 (3.7)	0.048
**Laparoscopic**			
No	95 (46.3)	9 (33.3)	
Yes	110 (53.7)	18 (66.7)	0.20
**Surgery for recurrence**			
No	145 (70.7)	20 (74.1)	
Yes	60 (29.3)	7 (25.9)	0.72

**Table 2:** Characteristics of patients in the non-IOUS and IOUS groups after propensity score matching analysis.

	Historical Cohort (*n* = 27)	IOUS (*n* = 27)	*p* Value
**Gender**			0.78
F	15 (55.6)	16 (59.3)	
M	12 (44.4)	11 (40.7)	
**Age (years)**			
Mean (SD)	40.4 (15.2)	38.3 (16.5)	0.61
**BMI**			
Mean (SD)	20.59 (5.14)	21.24 (2.03)	0.54
**Smoking**			
No	20 (74.1)	22 (81.5)	
Yes	7 (25.9)	5 (18.5)	0.51
**Stricturing disease**			
No	11 (40.7)	15 (55.6)	
Yes	16 (59.3)	12 (44.4)	0.28
**Penetrating disease**			
No	16 (59.3)	13 (48.1)	
Yes	11 (40.7)	14 (51.9)	0.41
**Localization**			
Ileal	8 (29.6)	8 (29.6)	
Ileocolonic	19 (70.4)	19 (70.4)	0.99
**Perianal disease**			
No	20 (74.1)	26 (96.3)	
Yes	7 (25.9)	1 (3.7)	0.02
**Laparoscopic**			
No	14 (51.9)	9 (33.3)	
Yes	13 (48.1)	18 (66.7)	0.17
**Surgery for recurrence**			
No	20 (74.1)	20 (74.1)	
Yes	7 (25.9)	7 (25.9)	0.99

**Table 3:** Outcomes of patients in the non-IOUS and IOUS groups after propensity score matching analysis.

	Historical Cohort (*n* = 27)	IOUS (*n* = 27)	*p* Value
**Histological margin**			0.021
No	14 (51.9)	22 (81.5)	
Yes	13 (48.1)	5 (18.5)	
**Length of specimen (cm)**			
Mean (SD)	34.1 (23.1)	24.1 (13.5)	0.058
**Length of surgery (minutes)**			
Mean (SD)	225.0 (77.8)	254.2 (49.3)	0.11

## Data Availability

The data underlying this article will be shared upon reasonable request to the corresponding authors.

## Abbreviations

The following abbreviations are used in this manuscript:

CD	Crohn’s disease
IOUS	Intraoperative ultrasound
IBD	Intestinal bowel disease
POR	Postoperative recurrence
TAB	Table
BMI	Body mass index
BWP	Bowel wall pattern
SD	Standard deviation
OR	ODD ratio
CI	Confidence interval
RR	Relative risk
HR	Hazard ratio
